# Supplementary material for: Altered microRNA Transcriptome in Cultured Human Liver Cells upon Infection with Ebola Virus
Source: Int J Mol Sci. 2021 Apr 6;22(7):3792. doi: 10.3390/ijms22073792 (PMC8038836; doi:10.3390/ijms22073792)
Supplement: Supplementary file 1 [file ijms-22-03792-s001.zip › Supplementary_File/C_ GO_Analysis_Results/16-30nt_go_Mayinga-24h-Huh7_vs_Control-24h-Huh7_down.mature_mirna_targets/CC_EnrichmentScore.pdf]

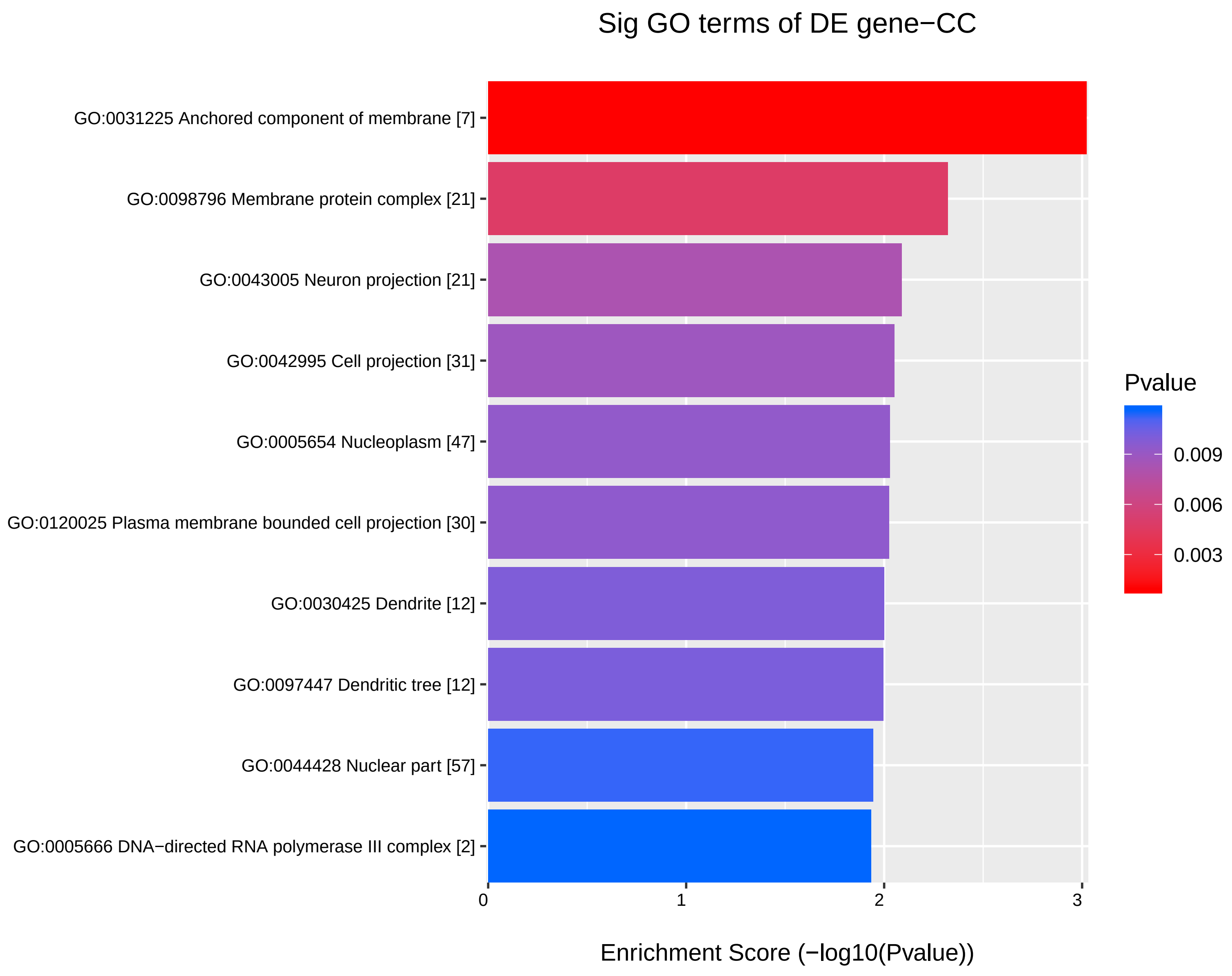

Sig GO terms of DE gene−CC
GO:0031225 Anchored component of membrane [7]
GO:0098796 Membrane protein complex [21]
GO:0043005 Neuron projection [21]
GO:0042995 Cell projection [31]
GO:0005654 Nucleoplasm [47]
GO:0120025 Plasma membrane bounded cell projection [30]
GO:0030425 Dendrite [12]
GO:0097447 Dendritic tree [12]
GO:0044428 Nuclear part [57]
GO:0005666 DNA−directed RNA polymerase III complex [2]
0
1
2
3
Enrichment Score (−log10(Pvalue))
Pvalue
0.009
0.006
0.003
